# Image enhancement of whole-body oncology [^18^F]-FDG PET scans using deep neural networks to reduce noise

**DOI:** 10.1007/s00259-021-05478-x

**Published:** 2021-07-28

**Authors:** Abolfazl Mehranian, Scott D. Wollenweber, Matthew D. Walker, Kevin M. Bradley, Patrick A. Fielding, Kuan-Hao Su, Robert Johnsen, Fotis Kotasidis, Floris P. Jansen, Daniel R. McGowan

**Affiliations:** 1 GE Healthcare, Big Data Institute, University of Oxford, Oxford, UK; 2 GE Healthcare, Waukesha, WI USA; 3 Oxford University Hospitals NHS FT, Oxford, UK; 4 Wales Research and Diagnostic PET Imaging Centre, University Hospital of Wales, Cardiff, UK; 5 Department of Radiology, University Hospital of Wales, Cardiff, UK; 6 GE Healthcare, Zurich, Switzerland; 7 Department of Oncology, University of Oxford, Oxford, UK

**Keywords:** Deep neural networks, PET, Image quality

## Abstract

**Purpose:**

To enhance the image quality of oncology [^18^F]-FDG PET scans acquired in shorter times and reconstructed by faster algorithms using deep neural networks.

**Methods:**

List-mode data from 277 [^18^F]-FDG PET/CT scans, from six centres using GE Discovery PET/CT scanners, were split into ¾-, ½- and ¼-duration scans. Full-duration datasets were reconstructed using the convergent block sequential regularised expectation maximisation (BSREM) algorithm. Short-duration datasets were reconstructed with the faster OSEM algorithm. The 277 examinations were divided into training (*n* = 237), validation (*n* = 15) and testing (*n* = 25) sets. Three deep learning enhancement (DLE) models were trained to map full and partial-duration OSEM images into their target full-duration BSREM images. In addition to standardised uptake value (SUV) evaluations in lesions, liver and lungs, two experienced radiologists scored the quality of testing set images and BSREM in a blinded clinical reading (175 series).

**Results:**

OSEM reconstructions demonstrated up to 22% difference in lesion SUV_max_, for different scan durations, compared to full-duration BSREM. Application of the DLE models reduced this difference significantly for full-, ¾- and ½-duration scans, while simultaneously reducing the noise in the liver. The clinical reading showed that the standard DLE model with full- or ¾-duration scans provided an image quality substantially comparable to full-duration scans with BSREM reconstruction, yet in a shorter reconstruction time.

**Conclusion:**

Deep learning–based image enhancement models may allow a reduction in scan time (or injected activity) by up to 50%, and can decrease reconstruction time to a third, while maintaining image quality.

**Supplementary Information:**

The online version contains supplementary material available at 10.1007/s00259-021-05478-x.

## Introduction

Positron emission tomography (PET) is a quantitative imaging modality that is used to study functional processes using specific radiotracers (e.g. metabolism using [^18^F]-fluorodeoxyglucose (FDG), prostate cancer detection using [^68^ Ga]-PSMA). The quality and quantitative accuracy of PET images are influenced by several factors such as scanner specifications (e.g. sensitivity, spatial resolution, timing resolution), patient demographics, imaging protocol (e.g. radiotracer, injected dose, post-injection delay, scan duration) and image reconstruction technique (e.g. point-spread-function modelling—PSF, convergence criteria, regularisation) [1].

For a given PET scanner, increasing the injected dose or scan time [2] and using advanced image reconstruction algorithms [3] will significantly improve the image quality in terms of noise and lesion detectability. On the other hand, there is a need to increase patient throughput and to reduce radiation dose; methods that allow for reduced scan time and/or injected dose without compromising the diagnostic accuracy of PET images can be important to achieve these goals. Advances in Bayesian iterative reconstruction techniques have led to improved quality of PET images by ensuring the reconstruction process considers all the statistical and physical processes involved during data acquisition. As a result, these reconstruction methods (e.g. ordered subsets expectation maximisation—OSEM and block sequential regularised expectation maximisation—BSREM [4]) have mostly superseded the analytic techniques in emission tomography—despite their computational burden. GE Healthcare’s commercial implementation of BSREM (Q.Clear) has found widespread clinical use [5].

Bayesian reconstruction algorithms aim to penalise the formation of noisy images based on the hypothesis that large local variations in voxel intensity in the images are likely due to noise. The strength of penalisation is explicitly controlled by a regularisation parameter (*β*) [6]. For a properly adjusted *β* value these techniques allow improved contrast to noise ratio (CNR) and lesion detectability [7]. For a zero *β*, the BSREM algorithm becomes an unregularised algorithm similar to OSEM, for which the number of iterations is often reduced in order to implicitly control noise, at the cost of reducing contrast and convergence. The major limitation of these hypothesis-driven algorithms is that, depending on their penalty function and the regularisation strength, they might suppress not only noise but also legitimate image features.

Deep learning (DL) techniques have recently been shown to have promising application in many aspects of PET imaging from photon detection to image reconstruction [8] and lesion detection [9]. For image reconstruction, deep convolutional neural networks (CNNs) have an immense potential to learn data-driven penalty functions that best represent noise and structures in the images and thus may address the limitation of the Bayesian reconstruction algorithms [10]. Recent DL developments in PET image reconstruction can be divided into three groups: (1) direct mapping of PET raw data (i.e. sinograms) to PET images using end-to-end networks [11–13]; (2) deep learning reconstruction (DLR), which combines DL with Bayesian reconstruction methods [14]; (3) deep learning enhancement (DLE) of PET images for noise reduction [15, 16] or improved convergence [17]. Direct methods aim to learn the whole reconstruction process from scratch; hence, their training is computationally intensive and requires big datasets, whereas DLR methods aim to merge the model-based Bayesian algorithms with CNNs to reduce their data requirement and computational burden. As DLE methods operate on the reconstructed images (noisy and/or partially converged), they can be trained and deployed using the current clinical workflow without re-architecting the reconstruction engines.

DLE methods have shown promising performance for image denoising [9] with comparable performance to DLR methods [14]. Moreover, image-based machine/deep learning techniques have enabled ultra-low dose PET scans maintaining clinically relevant information in terms of diagnostic accuracy and quantitative SUV measurements [18, 19]. The great potential for DLE motivated the current study, in which we trained and evaluated a DLE model. The goal was reduction of both patient scan time (or injected activity) and computational reconstruction time, while providing image quality at least comparable to an advanced Bayesian reconstruction method without the need for adjustment of any regularisation parameter. Specifically, the DLE was trained to map full- and partial-duration OSEM images (with TOF and PSF modelling) to full duration BSREM images. To the best of our knowledge, this study is the first that makes use of deep learning to produce BSREM-like images from conventional OSEM images.

## Materials and methods

**Fig. 1:**
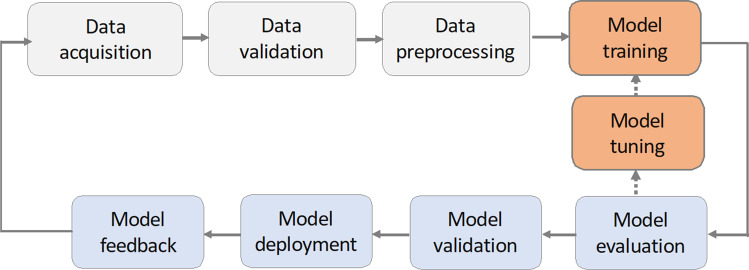
The pipeline and life cycle of our DLE model

Our DLE model was trained in supervised learning cycles for mapping low-contrast high-noise OSEM PET images to high-contrast low-noise BSREM ones in order to improve lesion detectability and diagnostic confidence of oncology [^18^F]-FDG scans. In this section, we elaborate on the steps involved in the life cycle of our model as illustrated in Fig. 1.

## Data acquisition, validation and pre-processing

The first step was to acquire PET list-mode data and CT-based attenuation correction (CTAC) images from multiple clinical sites to improve the generalisability of a DLE model to account for the fact that different sites use different PET/CT protocols (i.e. injected activity, uptake time, scan duration), have different oncology applications, and can have different scanner types and reconstruction parameters (e.g. BSREM’s beta value). A total of 277 whole-body oncology [^18^F]-FDG PET examinations were retrospectively collected from six clinical sites, each equipped with either GE Discovery MI (4 or 5 ring at 3 and 2 sites, respectively) or GE Discovery 710 PET/CT scanners (at one site), full details given in Table S1. The PET subsystems of these scanners have different spatial (4.8–4.9 mm at 1 cm) and TOF resolution (385–549 ps) as well as sensitivity depending on the number of detector rings and photodetection technology. Consequently, the [^18^F]-FDG activity (mean ± SD, 391 ± 136 MBq), scan duration (147 ± 8 s/bed) and regularisation parameter of BSREM reconstruction (beta 350–500, median 400) varied between sites. The [^18^F]-FDG uptake time varied between sites: 82 ± 28 min. Moreover, there were variations in disease and patient demographics (body mass index, BMI, 27.2 ± 6.0 kg/m^2^). As the model development was performed on fully anonymised image-only data from the six sites, a breakdown of their clinical conditions was not available. A table of these basic demographics for the 277 patients is shown in Table S1. For each subject, a whole-body CT scan was performed for PET attenuation correction using 100–120 kVp, 150–200 mAs.

The 277 examinations were validated for patient and tracer information and then divided into training (*n* = 237), validation (*n* = 15) and test (*n* = 25) sets. Each patient’s list-mode data was binned into four different duration sinograms including full, ¾, ½ and ¼ to simulate reduced scanning time (from the start of the scan to the given fraction). Each was reconstructed using an OSEM algorithm with a matrix size of 256 × 256, field-of-view 700 mm, voxel size 2.7 × 2.7 × (2.8 or 3.7) mm^3^ and 2 iterations, 34 and 24 subsets for Discovery MI and 710 scanners, respectively, with PSF and standard z-filter. Full duration sinograms were additionally reconstructed using the BSREM algorithm with a regularisation parameter that was experimentally adjusted per site in order to achieve the same low noise level (based on visual inspection) across data from all sites. The noisy, low-contrast OSEM and low-noise, high-contrast BSREM images were respectively used as input and target images for supervised training of our DLE model. The training and validation image volumes were pre-processed by cropping their matrix sizes by up to 40% (to save GPU memory) and were axially divided into equally spaced 3D sub-volumes (patches, of size 152 × 152 × 100). Using this multi-centre multi-duration training set, we aimed to train one generic DLE model capable of dealing with different noise levels and datasets.

## Model training and tuning

A 3D residual convolutional encoder–decoder (U-Net [20]) network was developed and implemented in PyTorch. As illustrated in Fig. S1, the network is composed of convolutional layers (using 3 × 3 × 3 kernels), batch normalisation (BN), 3D maximum pooling layers and tri-linear up-sampling layers, skip and residual connections and rectified linear unit (ReLU) activation functions. The network predicts a residue image (with positive and negative values) that is added to an input patch in order to reduce noise or improve the contrast of features. The network was trained in a supervised session in which its output (i.e. OSEM + DLE) is compared to a target patch (i.e. BSREM) based on a mean squared error (MSE) loss function (as a similarity measure) and then the resulting error is back propagated through the network using the Adam optimiser [21] to update its trainable parameters (i.e. convolution kernels, biases, BN parameters). In each training iteration (termed an epoch), the training patches were randomly shuffled. The network was trained for a maximum of 100 epochs on a workstation with two 24 GB RTX6000 GPUs, bridged via NVLink technology. The validation set was used to monitor the network’s generalisation error for inferencing on unforeseen datasets and to avoid over-fitting. The standardised uptake values (SUVs) of lesions, liver and lungs in the validation datasets were also used for model evaluation (as described in the next section). The epoch at which the model had the lowest validation loss, as well as the lowest feature SUV quantification differences with the target data, was chosen as a stopping criterion.

The performance of a DLE model is affected by many factors from data sufficiency and diversity to model architecture and training hyperparameters. Given the high computational load of training with large datasets, most of the model parameters were chosen experimentally (such as loss function, optimiser, learning rate, kernel size, batch size and patch dimensions). The model’s number of kernels was however based on quantitative measures and model feedback received from clinical review of the model’s output. Table S2 summarises three DLE models used in this study that differ mostly based on their number of trainable model parameters.

## Model evaluation, validation, deployment and clinical feedback

The performance of our trained DL models was objectively evaluated using the validation and test datasets in terms of lesion SUV_max_ (maximum voxel intensity), SUV_mean_ (mean intensity of voxels) in liver and lungs and the noise in the liver using volumes of interest (VOIs) selected per subject. For each subject, five VOIs of size 7 × 7 × 7 voxels were defined in the lungs, and five similar VOIs in liver. These were used for evaluation of SUV_mean_, the SD of noise and background variability. For each subject, up to five small lesions were visually identified and segmented using an adaptive thresholding method (42% of subtraction of maximum and minimum SUV in a 7 × 7 × 7 bounding box). The lung and liver VOIs were defined on target BSREM images and then transferred to the other image series of each exam (full- or partial-duration OSEM or DLE). The lesion segmentation was separately performed for each image series due to its adaptive thresholding basis. The difference in SUV values (compared to the target BSREM SUVs), scatter plots and Bland–Altman plots were used for objective evaluation of the results. The statistical significance of differences in SUV bias was also evaluated using the Wilcoxon signed-rank test.

At the evaluation stage, a model that was not over fitted (based on validation loss) and had the best quantification performance in lesions, liver and lungs for the validation set was chosen as the best model. Next in the model development, the selected model was evaluated using test datasets and was deployed for external evaluations outside of the training environment. Unlike a DLR model, DLE models can be readily deployed on local devices or on cloud-based AI inferencing due to their reduced computational burden and reduced data transfer requirements. While DLE are inferenced on only image data, which is very quick to transfer, DLR requires list data (or sinogram) as well as CT for attenuation correction. Based on initial clinical feedback, we had looped over the life cycle of the model and performed data versioning and model tuning (by changing the number of trainable parameters) in order to improve the models.

Two radiologists, reader 1 (K.M.B. 18 years board certified in clinical radiology and nuclear medicine) and reader 2 (P.A.F. 17 years board certified in clinical radiology and nuclear medicine), blinded to image reconstruction, independently rated all 25 PET/CT testing sets. Each of the 25 patient cases had 7 image series (full-duration BSREM, full-, ¾-, ½-duration OSEM and full-, ¾-, ½-duration DLE-standard); these were assessed based on Likert scores considering several image features (image quality, diagnostic confidence, noise, etc.). The Likert scale used was 0 (non-diagnostic), 1 (poor), 2 (satisfactory), 3 (good), 4 (very good) and 5 (excellent). In addition, the seven series were ranked in order of preference from 1 (best) to 7 (worst) for each imaging feature. If necessary two series were ranked equal, in this situation the two series were given the same rank with the following lower number subsequently missed in the ranking. To reduce the number of readings, the ¼-duration scans and DLE-smooth and sharp were not rated.

Inter-reader agreement was measured by using a quadratic weighted kappa (*κ*) [22], using SPSS version 27. The statistical significance of differences in image quality score was evaluated using the Wilcoxon signed-rank test between the target BSREM reconstruction and DLE-standard for each frame duration.

## Results

**Fig. 2:**
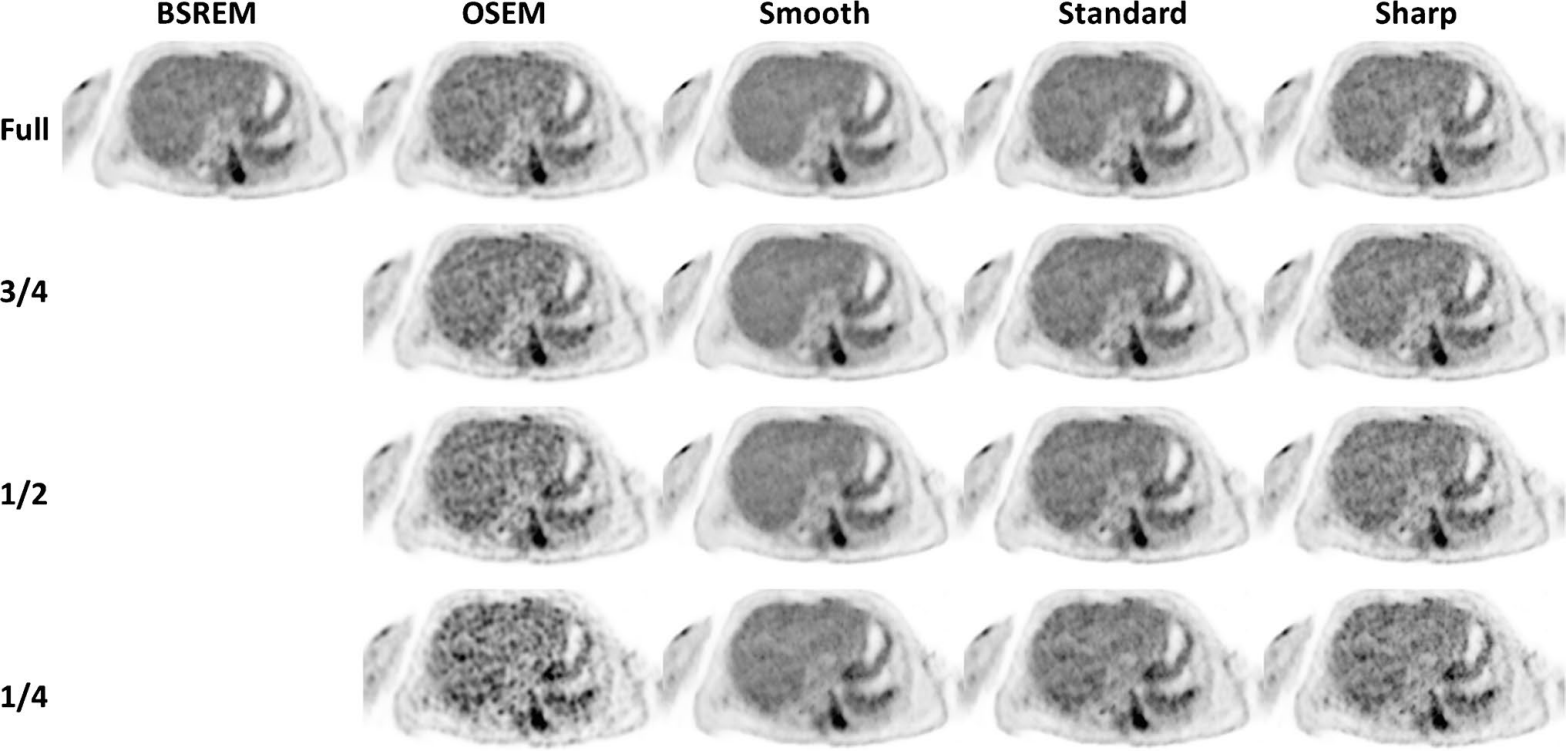
Visual comparison of different reconstruction methods and deep learning enhancement (DLE) models for different scan durations of a representative subject with a BMI of 35.0 kg/m^2^ with an injected activity of 222 MBq scanned on GE Discovery MI (4-ring) PET/CT scanner (slice thickness 2.8 mm). BSREM = block sequential regularised expectation maximisation

Figure 2 compares the results of OSEM and DLE methods for different scan durations of a representative patient compared to its target full-duration BSREM. The results show liver noise in OSEM is increased as scan duration is decreased, while smooth, standard and sharp DLEs show consistently reduced noise (at different levels depending on the DLE model). Based on initial clinical reading, DLE-standard was found to be a model that provides the level of enhancement that is comparable to BSREM (with medium level of regularisation). In the following, we report the results for DLE-standard. Corresponding results for DLE-smooth and sharp are presented in the Supplementary materials.

**Fig. 3:**
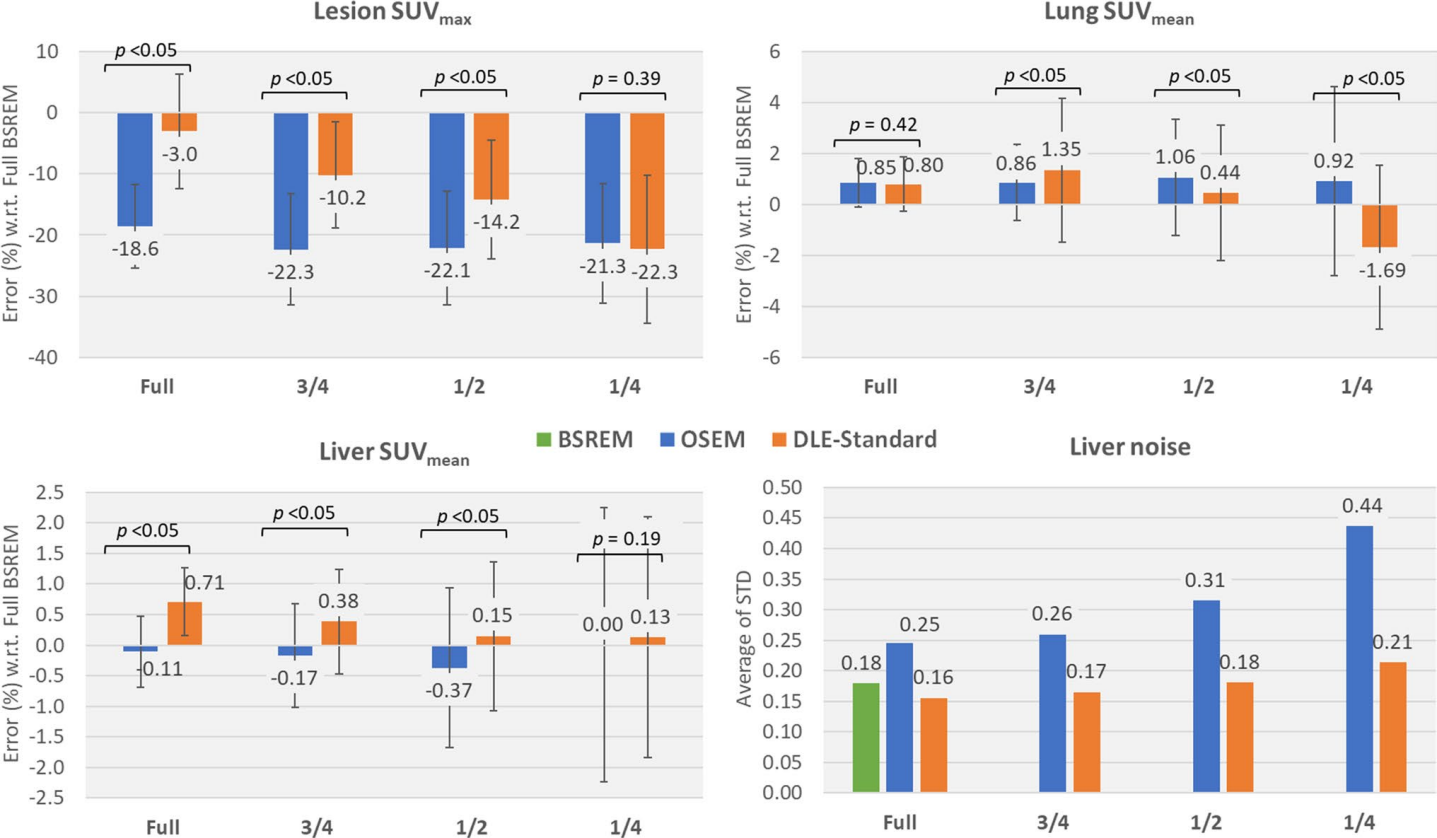
Quantitative performance of the DLE-standard model evaluated on the testing set in terms of lesion SUV_max_, lung SUV_mean_, liver SUV_mean_ and noise in liver for full-, ¾-, ½- and ¼-duration input scans. Average of STD is the SD of noise averaged over all 5 liver VOIs across all patients. The *p* values shown are calculated using the Wilcoxon signed-rank test from the differences in SUV bias (OSEM to DLE-standard). BSREM = block sequential regularised expectation maximisation, DLE = deep learning enhancement

Figure 3 shows the quantitative performance of OSEM and DLE-standard methods on the testing set for lesion SUV_max_, lung and liver SUV_mean_, and liver noise for full- and partial-duration scans in comparison with the target BSREM method. Figures S2 and S3 show the corresponding results for DLE-smooth and DLE-sharp. The *p* values on the figures show the significance of the difference between DLE and OSEM results for each scan duration. Table S3 shows the *p* values of the difference between lesion SUV_max_, lung and liver SUV_mean_ for each reconstruction method and the full-duration BSREM for all scan durations. The OSEM method shows a higher percentage difference in SUV_max_ of lesions (*n* = 67) as compared to full-duration BSREM images (up to 22%), due to lack of convergence, and higher noise level in the liver, due to lack of explicit regularisation. DLE-standard shows lower SUV_max_ quantification differences for lesions at different scan durations down to half duration. In the lung and liver, we found that both OSEM and DLE-standard had minimal bias in SUV_mean_ as the total counts in the image was decreased; changes were around 2% or less which is of little clinical significance. The liver noise is relatively similar for all scan durations; however, the lesion quantification errors become larger as the scan duration is reduced. This can be attributed to the fact that DLE acts like a BSREM reconstruction with a beta value that is automatically increased for shorter or noisier scans, reducing noise at the cost of a reduction in contrast (and lesion SUV_max_). As shown in Figs. S2 and S3, DLE-smooth behaves similarly to DLE-standard however with a lower noise in liver, whereas DLE-sharp shows less noise reduction and better lesion quantification performance.

**Fig. 4:**
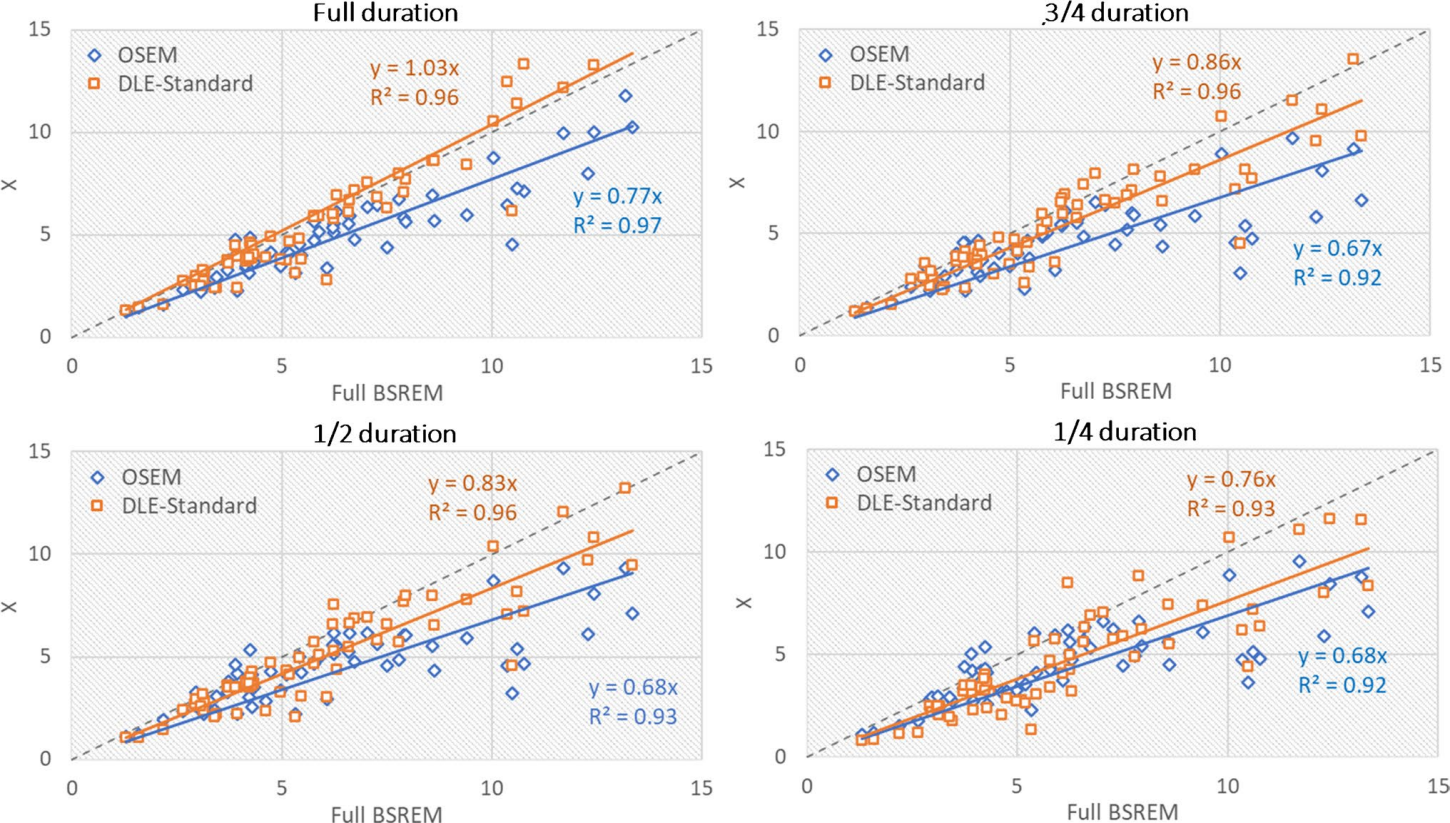
Scatter plots of lesion SUV_max_ for different durations of OSEM and DLE-standard images compared to full-duration BSREM images. The grey line is an identity line. BSREM = block sequential regularised expectation maximisation, DLE = deep learning enhancement

Figure 4 shows scatter plots of lesion SUV_max_ for different duration OSEM and DLE-standard images compared to full-duration BSREM images. Figures S4 and S5 show similar results for DLE-smooth and sharp, respectively. As shown in Fig. 4, for full-duration scans, the slope of DLE-standard is close to identity (gradient = 1.03), which indicates contrast convergence enhancement of the input OSEM images (which yielded a gradient of 0.77). For partial-duration scans, DLE provides less enhancement in terms of the slope of the fitted lines, but with more noise reduction as shown in Fig. 3. Similar trends are observed for DLE-smooth and sharp.

**Fig. 5:**
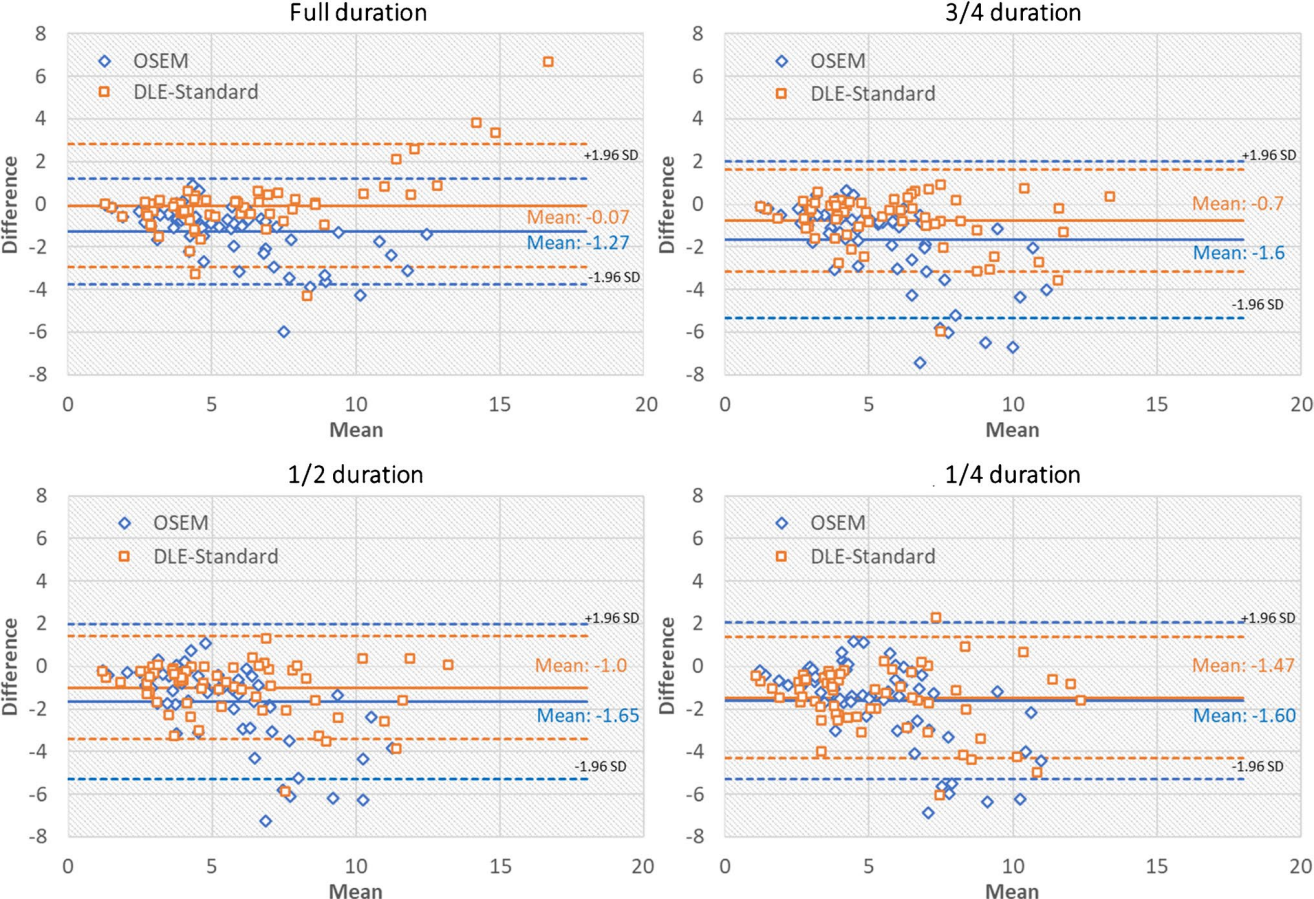
Bland–Altman plots comparing the concordance of lesion SUV_max_ between full-duration BSREM and different durations of the OSEM and DLE-standard. Actual values for limits of agreement are shown in Table S4 for clarity. BSREM = block sequential regularised expectation maximisation, DLE = deep learning enhancement

Figure 5 shows Bland–Altman plots comparing the concordance of lesion SUV_max_ between full-duration BSREM and different durations of the OSEM and DLE-standard. Figures S6 and S7 show similar results for DLE-smooth and sharp, respectively. Table S4 summarises the means and the limits of agreement (1.96 SDs) for all DLEs and scan durations. Consistent with the other quantification measures, the plots show a systematic difference in SUV_max_ between OSEM and DLE methods for full-duration scans, with this difference reducing as the duration reduces from full to ¼-duration scans.

**Fig. 6:**
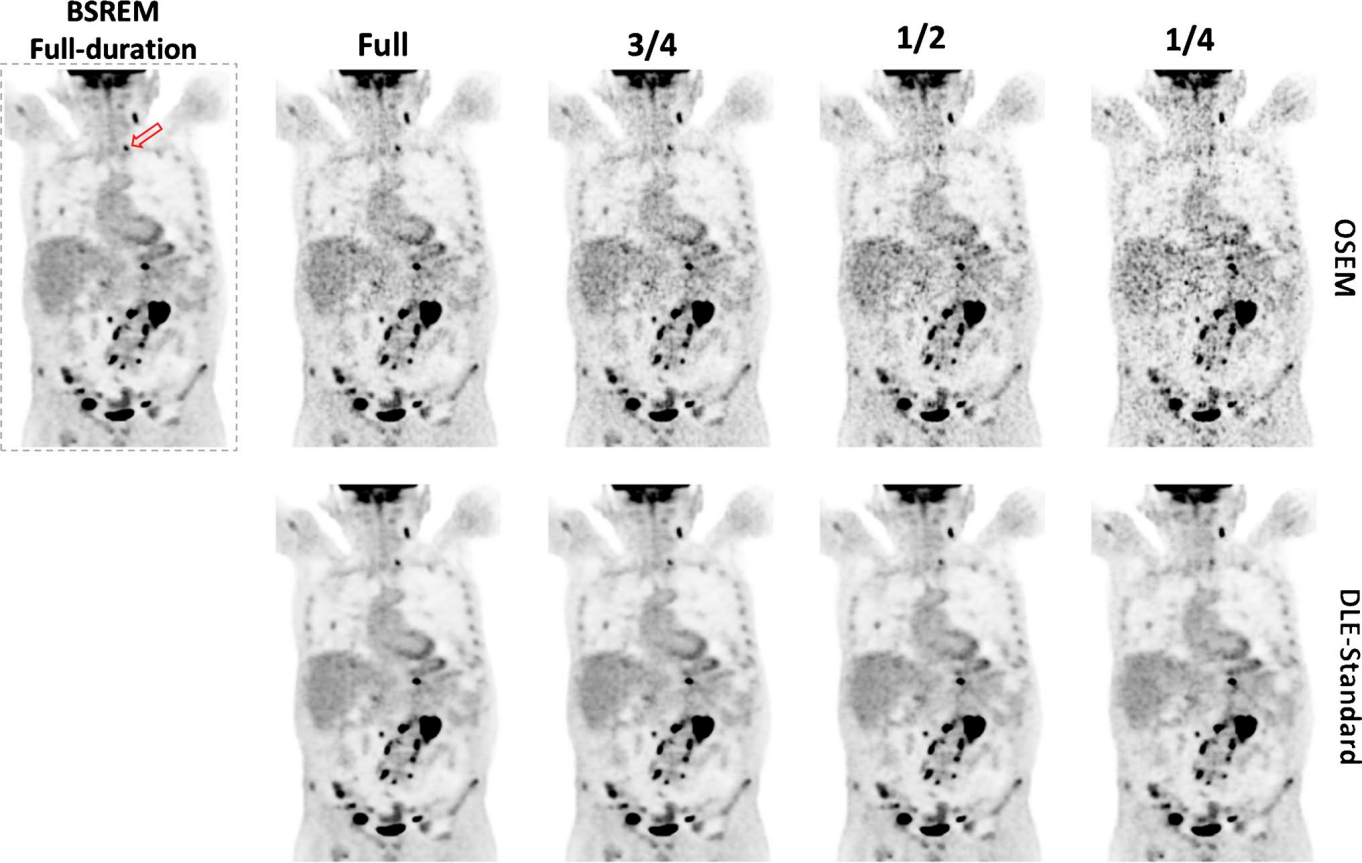
Reconstruction results for a patient with a BMI of 23.9 kg/m^2^ with an injected activity of 289 MBq scanned on GE D710 PET/CT scanner (slice thickness 3.7 mm). This patient had a history of relapsed DLBCL (diffuse large B-cell lymphoma). Their blood glucose was 7.8 mmol/l. The arrow points to a small pathological sub-centimetre node at the root of the left side of the neck. BSREM = block sequential regularised expectation maximisation, DLE = deep learning enhancement

Figure 6 shows the reconstruction results for an [^18^F]-FDG scan for DLE-standard. Figure S8 shows similar results for DLE-smooth and sharp, respectively. As shown, the DLE-standard model produces images of consistent noise and contrast similar to the target images. It is noteworthy that such a noise reduction level does not adversely impact lesion detectability down to ½-duration scans. For ¼-duration scans, some lesions or features have low signal-to-noise ratio and manifest similar to noise. While DLE-sharp is likely to preserve such features (with less noise reduction), DLE-smooth is likely to suppress them.

Tables 1, 2 and 3 show the scores and ranking results for different reconstruction methods. During clinical scoring, both radiologists reported that the images scored covered a broad range of oncological conditions. No images were scored non-diagnostic. The results show that short scans with DLE filtering provide a score comparable or higher than BSREM for all imaging metrics. Testing the significance between (full-duration) BSREM and DLE-standard for image quality score showed a significant preference for DLE over BSREM when the DLE input was full-duration (*p* < 0.001) or ¾-duration scans (*p* = 0.006). While full DLE-standard and ¾ DLE-standard showed significant superiority, there was no significant difference found for ½ DLE-standard (*p* = 0.11). These ½-time DLE images had slightly lower (inferior) average image quality scores compared to full-time BSREM, and the absence of statistical significance in this test does not demonstrate clinical equivalence. From average rankings, full DLE-standard was preferred followed by ¾ DLE-standard and then BSREM for all imaging metrics.

**Table 1:** Clinical image quality scoring from two readers of 25 whole-body scans based on different criteria, mean (1 SD)

Scores	Image quality	Liver IQ	Bone marrow IQ	Background tissues except liver/marrow	Noise level	Lesion detectability	Diagnostic confidence
Full BSREM	3.6 (0.81)	3.4 (0.79)	3.7 (0.85)	3.6 (0.84)	3.5 (0.79)	4.0 (0.83)	3.9 (0.88)
Full OSEM	2.7 (0.78)	2.6 (0.75)	2.9 (0.87)	2.7 (0.80	2.7 (0.77)	2.9 (0.72)	2.9 (0.71)
¾ OSEM	2.6 (0.67)	2.6 (0.67)	2.9 (0.93)	2.7 (0.77)	2.6 (0.67)	2.7 (0.64)	2.6 (0.64)
½ OSEM	1.9 (0.70)	1.8 (0.64)	2.1 (0.85)	1.9 (0.77)	1.8 (0.71)	1.9 (0.75)	1.9 (0.75)
Full DLE-standard	**4.2 (0.84)**^a^	**4.1 (0.84)**	**4.3 (0.78)**	**4.2 (0.80)**	**4.1 (0.81)**	**4.4 (0.76)**	**4.4 (0.67)**
¾ DLE-standard	3.9 (0.63)^b^	3.8 (0.65)	4.0 (0.65)	3.9 (0.63)	3.9 (0.63)	3.9 (0.78)	4.0 (0.71)
½ DLE-standard	3.3 (0.79)^c^	3.4 (0.64)	3.7 (0.75)	3.5 (0.65)	3.4 (0.64)	3.2 (0.95)	3.4 (0.92)
Weighted kappa	0.62	0.66	0.60	0.51	0.51	0.52	0.62

0 is non-diagnostic, 5 is excellent with no or minimal heterogeneities. Bold indicates the best (highest) score for each metric. Quadratically weighted kappa values between the two readers are given for each metric. The image quality scores from DLE were also tested for significant differences as compared to full BSREM

^a^*p* < 0.001, ^b^*p* = 0.006, ^c^*p* = 0.11

**Table 2:** Clinical image quality ranking from two readers of 25 whole-body scans based on different criteria, mean (1 SD)

Ranks	Image quality	Liver IQ	Bone marrow IQ	Background tissues except liver/marrow	Noise level	Lesion detectability	Diagnostic confidence
Full BSREM	2.6 (1.3)	2.6 (1.3)	2.6 (1.3)	2.6 (1.3)	2.6 (1.2)	2.3 (1.23)	2.4 (1.2)
Full OSEM	5.0 (1.2)	5.1 (1.0)	5.0 (1.2)	5.1 (1.0)	5.1 (1.0)	4.5 (1.19)	4.8 (1.1)
¾ OSEM	5.3 (0.99)	5.4 (0.86)	5.2 (1.3)	5.4 (0.85)	5.4 (0.85)	5.1 (0.99)	5.4 (0.86)
½ OSEM	6.8 (0.42)	6.8 (0.48)	6.7 (0.95)	6.8 (0.49)	6.8 (0.48)	6.8 (0.45)	6.9 (0.36)
Full DLE-standard	**1.3 (0.87)**	**1.2 (0.68)**	**1.2 (0.68)**	**1.2 (0.68)**	**1.2 (0.68)**	**1.5 (0.91)**	**1.5 (0.91)**
¾ DLE-standard	1.8 (0.83)	1.6 (0.72)	1.6 (0.73)	1.6 (0.73)	1.6 (0.72)	2.2 (1.21)	2.0 (0.82)
½ DLE-standard	3.4 (1.5)	2.9 (1.4)	2.6 (1.4)	2.9 (1.4)	3.0 (1.4)	4.1 (1.91)	3.8 (1.57)

1 is best and 7 is worst. Bold indicates the best (lowest) rank for each metric

Apart from the improved contrast-to-noise ratio, DLE can mimic BSREM reconstruction with a lower overall computational burden. The reconstruction time of BSREM and OSEM algorithms on a 5-ring DMI scanner are 3.2 and 1.1 min per bed position, respectively, with DLE additional processing for a whole-body scan only ~ 5 s. Therefore, DLE can provide an image quality comparable to BSREM in almost one third of the reconstruction time.

**Table 3:** Percentage of clinical image quality scores greater than or equal to 3 from two readers of 25 whole-body scans based on different criteria

Scores	Image quality	Liver IQ	Bone marrow IQ	Background tissues except liver/marrow	Noise level	Lesion detectability	Diagnostic confidence
Full BSREM	92	88	96	92	92	96	96
Full OSEM	40	36	56	40	40	52	52
¾ OSEM	48	40	52	48	48	40	40
½ OSEM	4	0	12	8	4	8	4
Full DLE-standard	**100**	**100**	**100**	**100**	**100**	**100**	**100**
¾ DLE-standard	**100**	**100**	**100**	**100**	**100**	96	96
½ DLE-standard	92	96	**100**	**100**	96	88	88

Bold indicates the best (highest) percentage for each metric

## Discussion

In the present study, the feasibility of generalised deep learning denoising model development was explored, and subsequently the performance of three selected models was evaluated. These models aim to facilitate a substantial reduction in scan duration together with a reduced reconstruction/processing time for whole-body [^18^F]-FDG PET scans compared to the BSREM algorithm. These DLEs had a good performance on a range of metrics, for input data of different scan durations, from multiple PET centres, for a range of patient BMIs and across a spectrum of oncology indications. Crucially, the DLEs have no need for the user to fine tune the level of denoising or contrast enhancement per patient (or patient group).

Given that the ideal level of smoothness is subjective, we trained three different models for smooth, standard and sharp DLEs, among which the standard model was selected for further clinical evaluation. Our quantitative results showed that DLE-standard achieved a balanced performance for lesion detection, as well as lung and liver background noise compared to our target full-duration BSREM images (for which the regularisation parameter had been adjusted to provide a standard smoothness level). We found the DLE-sharp provided the best performance in terms of lesion SUV_max_ quantification (vs. target BSREM), as it incorporated the least noise reduction. Such behaviour was predictable as the models were trained to mimic BSREM images which have the same trade-off between noise and contrast as the regularisation parameter is changed. In order to reduce noise in short scans, the beta value of BSREM must be increased which comes at the cost of lowering SUV_max_ compared to full-duration BSREM. The ½-duration DLE-sharp had an error of 4.9% on lesion SUV_max_ compared to full-duration BSREM, whereas ½-duration DLE-standard had an error of 14.2%. Hence, if DLE is applied to achieve an image quantitatively similar to BSREM, then the DLE-sharp model should be chosen.

This study utilised a U-Net model as a widely used encoder–decoder CNN. Lu et al. [15] showed that an optimised 3-D U-Net can outperform a convolutional autoencoder network and a generative adversarial net for lung nodule quantification in reduced dose scans. The performance and generalisability of supervised deep learning models also depend on the quality and diversity of the training sets. For this reason, unsupervised DL models have also been explored for denoising of PET data [23]. However, their performance will instead depend on the network architecture and its training hyperparameters. For this reason, supervised DL methods trained with sufficient datasets are potentially more robust than unsupervised ones. In this study, we additionally targeted improved lesion contrast which can be challenging to be achieved by unsupervised methods. The relationship between the number of parameters in our U-net model and the level of sharpness relies on the fact that the U-net has a bottle neck where it compresses all semantic features. Based on our experience, the larger that bottle neck (hence the more parameters), the more details and noise are preserved. The smoothness behaviour of our networks was hence controlled by changing the number of such parameters.

A key strength of these current DLE models is the information variety of the training sets across different scanner types, acquisition protocols and patient demographics. This not only makes the approach applicable for a wide range of clinical situations but also eliminates the need for finding a set of good reconstruction parameters for each patient (e.g. the number of iterations, the strength of post-filtering, the beta value of BSREM recon, etc.). In fact, our clinical reading results in Tables 1 and 2 demonstrated for short scans DLE can provide a comparable or higher image quality than full-duration BSREM. This can be attributed to the fact that the regularisation parameter (beta) of BSREM is often selected per site or protocol rather than per patient, while with DLE a regularisation step suitable for the image set at hand is performed by the trained convolutional neural networks.

In comparison to DLE, the level of noise in BSREM reconstructions must be controlled using a regularisation parameter which is usually optimised per site or tracer or protocol. In a [^68^Ga]-DOTA PET study [24], it was found that adapting the regularisation parameter enables a one-third reduction of acquisition time or injected activity. Moreover, DLE models can be deployed across different PET scanners across different sites via cloud-based inferencing services. However, the new generation of long axial FOV and digital scanners combined with optimised reconstruction allow for ultra-fast imaging (i.e. 30 s per bed) [25]. In that regard, a DLE model trained on sufficiently diverse datasets could be used across all generations of PET scanners.

He et al. [17] utilised a DLE method to reduce the reconstruction time of BSREM algorithm through using less iterations to achieve full convergence (known as leapfrogging). In comparison, in this study, the ‘leapfrogging’ and faster reconstruction was achieved by transforming an OSEM reconstruction, which uses a small number of iterations, into fully converged BSREM-like images, which uses a large number of iterations. Our results demonstrate that the reconstruction time can thus be reduced, potentially to a third.

The present study has a number of limitations. The proposed method was not compared to other classical denoising methods such as non-local means (NLM). Such methods are however often hypothesis-driven with their performance limited by their mathematical form or their hyperparameters. For instance, NLM has a shape hyperparameter that governs its noise reduction and edge preservation. Since the present study included a large number of test sets, each with three noise levels, we decided to focus on the evaluation and deployment of DLE models, given their noise reduction and leapfrogging potential. The models were developed using data from three types of GE PET/CT scanner (D710, DMI 4-ring and DMI 5-ring). Currently, the only available commercial implementation of BSREM for PET is by GE Healthcare. The model development and its current validation were hence limited to images from a single scanner manufacturer where the necessary BSREM images were available. In addition, future work should incorporate a prospective study to include lesion detectability to provide greater certainty on this specific aspect of performance. Such a study is anticipated once the DLE algorithms can be readily deployed to sites. We can however be guided by the results from clinical scoring (Table 1) in which full DLE-standard had a higher lesion detectability, and ¾ DLE-standard a similar lesion detectability, as compared to full BSREM. Conversely, the ½ DLE-standard had a lower lesion detectability than full BSREM, while still being higher than full OSEM. The lesion detectability for all durations is expected to be higher when DLE-sharp is used. Another limitation is that in the lesion selection process for quantitative analysis, most of the identified lesions in our testing set had SUV_max_ > 2.5. Future work could be performed to study the quantitative performance of our DLE models for lesions SUV_max_ < 2.5. Such lesions were, however, present in the 25 cases scored by the two raters in this study. The performance of these DLE models (or similar ones) on whole-body dynamic or gated scans (which often provide noisy images), as well as on non-[^18^F]-FDG tracers, will also be of interest in the future. Future work will extend the training and testing of DLE with PET data from clinical sites not included in this current work, which will further evaluate and develop the DLE model. Future work could also use new advanced and computationally expensive reconstruction algorithms to train further AI algorithms as the clinically orientated time constraint is removed in this situation.

## Conclusion

This study developed deep learning image enhancement models for denoising and/or contrast image enhancement of full- and short-duration [^18^F]-FDG PET scans. Our quantitative and qualitative results demonstrate that with the proposed networks, that scan time or injected activity can be reduced by at least 25% versus BSREM and 50% versus OSEM without impacting the image quality. The networks predicted patient-specific BSREM-type images with a threefold faster reconstruction/processing time and without the need for tuning a regularisation parameter. Since the image quality and noise preference is subjective, three different models were trained and tested. The current evaluation found DLE to provide image quality at least as good as the full-duration BSREM reconstruction.

## Supplementary Information

Below is the link to the electronic supplementary material.

Supplementary file1 (DOCX 2937 KB)

## Data Availability

Data is available under reasonable request to the corresponding author.

## Code availability

The code used in the preparation of this paper is not available for public distribution.
