# Supplementary material for: Image enhancement of whole-body oncology [18F]-FDG PET scans using deep neural networks to reduce noise
Source: Eur J Nucl Med Mol Imaging. 2021 Jul 28;49(2):539–49. doi: 10.1007/s00259-021-05478-x (PMC8803788; doi:10.1007/s00259-021-05478-x)
Supplement: Supplementary file 1 — Supplementary file1 (DOCX 2937 KB) [file 259_2021_5478_MOESM1_ESM.docx]

**Supplementary Materials**

**Fig 1.** The schematic architecture of the DLE network used in this study. The number of kernels for DLE smooth in the encoder is 32, 64, 128, 256, for DLE standard is 42, 84, 168, 336, for DLE sharp is 64, 128, 512, 1024. See Supp. Table 2 for other details as well as Ref [20].

**Fig 2.** Quantitative performance of the smooth DLE model evaluated on the testing set in terms of lesion SUV_max_, lung SUV_mean_, Liver SUV_mean_ and noise in liver for full-, ¾-, ½- and ¼- duration input scans. Average of STD is the standard deviation of noise averaged over all 5 liver VOIs across all patients.

**Fig 3.** Quantitative performance of the sharp DLE model evaluated on the testing set in terms of lesion SUV_max_, lung SUV_mean_, Liver SUV_mean_ and noise in liver for full-, ¾-, ½- and ¼- duration input scans. Average of STD is the standard deviation of noise averaged over all 5 liver VOIs across all patients.

**Fig 4.** Scatter plots of lesion SUV_max_ for different duration of OSEM and DLE-smooth images compared to full-duration BSREM images. The grey line is an identity line.

**Fig 5.** Scatter plots of lesion SUV_max_ for different duration of OSEM and DLE-sharp images compared to full-duration BSREM images. The grey line is an identity line.

**Fig 6.** Bland-Altman plots comparing the concordance of the SUV_max_ of lesion between full-duration BSREM and different durations of the OSEM and DLE-smooth. Actual values for limits of agreement are shown in Supp. Table 4 for clarity.

**Fig 7.** Bland-Altman plots comparing the concordance of the SUV_max_ of lesion between full-duration BSREM and different durations of the OSEM and DLE-sharp. Actual values for limits of agreement are shown in Supp. Table 4 for clarity.

**Fig 8**. An extension of Fig.6 in the main manuscript for smooth and sharp DLEs. Reconstruction results for a patient with a BMI of 23.9 kg/m^2^ with an injected activity of 289 MBq scanned on GE D710 PET/CT scanner (slice thickness 3.7 mm). This patient had a history of relapsed DLBCL (Diffuse Large B-Cell Lymphoma). Their blood glucose was 7.8 mmol/l. The arrow points to a small pathological sub-cm node at the root of the left side of the neck.

**Table 1.** Demographic details for the patient studies used in the training, validation and testing of DLE. [SD=standard deviation]

|  | | Training | Testing | Validation |
| --- | --- | --- | --- | --- |
| Activity | Mean | 400 | 387 | 493 |
|  | SD | 217 | 131 | 66.6 |
|  | Median | 396 | 384 | 515 |
| Height | Mean | 1.72 | 1.70 | 1.74 |
|  | SD | 0.10 | 0.080 | 0.088 |
|  | Median | 1.72 | 1.71 | 1.75 |
| Weight | Mean | 79.4 | 77.8 | 96.5 |
|  | SD | 20.0 | 17.5 | 24.7 |
|  | Median | 76.0 | 77.0 | 92.0 |
| BMI | Mean | 26.9 | 26.9 | 32.0 |
|  | SD | 5.87 | 6.00 | 7.46 |
|  | Median | 25.6 | 26.4 | 31.6 |
| Number of slices | Mean | 413.5 | 413 | 446 |
|  | SD | 134.0 | 131 | 153 |
|  | Median | 345.0 | 345 | 345 |
| Number of D710 patients | | 29 | 0 | 0 |
| Number of DMI 4 ring patients | | 79 | 10 | 4 |
| Number of DMI 5 ring patients | | 129 | 15 | 11 |
| Number of clinical sites | | 5 | 4 | 3 |
| Number of site 1 patients | | 29 | 0 | 0 |
| Number of site 2 patients | | 22 | 3 | 3 |
| Number of site 3 patients | | 57 | 7 | 0 |
| Number of site 4 patients | | 0 | 0 | 1 |
| Number of site 5 patients | | 50 | 5 | 0 |
| Number of site 6 patients | | 79 | 10 | 11 |

**Table 2.** Model specifications and training parameters used in this study. Smooth, standard and sharp provide different levels of smoothness in the resulting images. [MSE=mean square error]

| DLE Model | No. trainable parameters | No. kernels in 1^st^ layer | No. max pooling layers | Kernel size | Batch size | Learning rate | No. epochs | Optimiser | Loss function |
| --- | --- | --- | --- | --- | --- | --- | --- | --- | --- |
| Smooth | 10,043,073 | 32 | 4 | 3×3×3 | 8 | 0.005 | 100 | Adam | MSE |
| Standard | 17,297,953 | 42 | 4 | 3×3×3 | 8 | 0.008 | 100 | Adam | MSE |
| Sharp | 40,158,593 | 64 | 4 | 3×3×3 | 8 | 0.001 | 100 | Adam | MSE |

**Table 3**. The p-values of the difference between features for each reconstruction method and the full-duration BSREM for all scan durations.

| Recon | Feature | Full | ¾ | ½ | ¼ |
| --- | --- | --- | --- | --- | --- |
| OSEM | Lesion SUV_max_ | <0.001 | <0.001 | <0.001 | <0.001 |
|  | Lung SUV_mean_ | <0.001 | 0.0028 | 0.026 | 0.54 |
|  | Liver SUV_mean_ | 0.30 | 0.29 | 0.18 | 0.71 |
| DLE-smooth | Lesion SUV_max_ | 0.099 | <0.001 | <0.001 | <0.001 |
|  | Lung SUV_mean_ | <0.001 | <0.001 | <0.001 | <0.001 |
|  | Liver SUV_mean_ | <0.001 | <0.001 | <0.001 | <0.001 |
| DLE-standard | Lesion SUV_max_ | 0.10 | <0.001 | <0.001 | <0.001 |
|  | Lung SUV_mean_ | <0.001 | 0.050 | 0.69 | 0.0031 |
|  | Liver SUV_mean_ | <0.001 | 0.0025 | 0.26 | 0.34 |
| DLE-sharp | Lesion SUV_max_ | 0.058 | 0.13 | 0.013 | <0.001 |
|  | Lung SUV_mean_ | <0.001 | <0.001 | <0.001 | 0.15 |
|  | Liver SUV_mean_ | <0.001 | <0.001 | <0.001 | 0.0013 |

**Table 4.** The mean and limits of agreement (1.96SD) values for all DLEs and scan durations from the Bland-Altman plots in Fig 5 in the main manuscript and supplementary Figs. 6 and 7.

| **Scan Duration** | **Reconstruction** | **+1.96 SD** | **mean** | **-1.96SD** |
| --- | --- | --- | --- | --- |
| **Full** | OSEM | 1.19 | -1.28 | -3.75 |
|  | DLE-Smooth | 1.78 | -0.14 | -2.07 |
|  | DLE-Standard | 2.81 | -0.07 | -2.95 |
|  | DLE-Sharp | 2.87 | -0.23 | -2.34 |
| **3/4** | OSEM | 2.02 | -1.66 | -5.35 |
|  | DLE-Smooth | 2.04 | -0.57 | -3.19 |
|  | DLE-Standard | 1.61 | -0.77 | -3.15 |
|  | DLE-Sharp | 2.49 | -0.38 | -3.27 |
| **1/2** | OSEM | 1.98 | -1.66 | -5.30 |
|  | DLE-Smooth | 1.91 | -0.90 | -3.71 |
|  | DLE-Standard | 1.41 | -1.00 | -3.42 |
|  | DLE-Sharp | 2.22 | -0.54 | -3.31 |
| **1/4** | OSEM | 2.07 | -1.60 | -5.28 |
|  | DLE-Smooth | 1.77 | -1.56 | -4.90 |
|  | DLE-Standard | 1.36 | -1.48 | -4.32 |
|  | DLE-Sharp | 2.23 | -0.75 | -3.74 |
